# *Clostridium butyricum MIYAIRI 588* Reduces Colorectal Adenomatous Polyp Recurrence: A Randomized Crossover Trial

**DOI:** 10.32604/or.2025.070432

**Published:** 2025-11-27

**Authors:** Jiunn-Wei Wang, Wen-Hung Hsu, Fang-Jung Yu, Fu-Chen Kuo, Chung-Jung Liu, Chao-Hung Kuo, Jaw-Yuan Wang, Ming-Hong Lin, Deng-Chyang Wu

**Affiliations:** 1 Graduate Institute of Clinical Medicine, College of Medicine, Kaohsiung Medical University, Kaohsiung, 807378, Taiwan; 2 Department of Internal Medicine, Faculty of Medicine, College of Medicine, Kaohsiung Medical University, Kaohsiung, 807378, Taiwan; 3 Division of Gastroenterology, Department of Internal Medicine, Kaohsiung Medical University Hospital, Kaohsiung, 80756, Taiwan; 4 Division of Gastroenterology, Kaohsiung Medical University Gangshan Hospital, Kaohsiung, 820111, Taiwan; 5 School of Medicine, College of Medicine, E-Da Hospital, I-Shou University, Kaohsiung, 84001, Taiwan; 6 Regenerative Medicine and Cell Therapy Research Center, Kaohsiung Medical University, Kaohsiung, 807378, Taiwan; 7 Department of Surgery, Faculty of Medicine, College of Medicine, Kaohsiung Medical University, Kaohsiung, 807378, Taiwan; 8 Division of Colorectal Surgery, Department of Surgery, Kaohsiung Medical University Hospital, Kaohsiung, 80756, Taiwan; 9 Department of Microbiology and Immunology, College of Medicine, Kaohsiung Medical University, Kaohsiung, 807378, Taiwan

**Keywords:** Gut microbiota, *Clostridium butyricum*, probiotics, colorectal polyps, adenoma prevention

## Abstract

**Objectives:**

Colorectal adenomatous polyps frequently recur after removal and are precursors to colorectal cancer, highlighting the need for effective preventive strategies. This study evaluated the efficacy of probiotic *Clostridium butyricum MIYAIRI 588* (CBM588) in preventing colorectal adenoma recurrence in high-risk patients.

**Methods:**

We conducted a randomized, single-blind, two-year crossover trial in patients with a history of adenomatous polyps. Participants received CBM588 in either the first or second year, with the alternate year as observation, and underwent annual surveillance colonoscopies. Outcomes (adenoma recurrence and polyp counts) were analyzed by intention-to-treat (ITT) and per-protocol (PP) approaches.

**Results:**

A total of 398 participants were enrolled. In first-year ITT analysis, the CBM588-first group had a lower mean polyp count (0.78 vs. 1.00) and lower adenoma recurrence rate (30.00% vs. 35.35%) than the control-first group, though these differences were not statistically significant (*p* = 0.10 and *p* = 0.26, respectively). In contrast, first-year PP analysis showed significant reductions in the CBM588-first group’s mean polyp count (0.80 vs. 1.25, *p* < 0.05) and adenoma recurrence rate (29.76% vs. 44.71%, *p* < 0.05) compared to control. After crossover, the group receiving CBM588 in the second year experienced similar benefits, and by year two both groups had comparable outcomes. No carryover effect was evident. The number needed to treat to prevent one adenoma in one year was 19 in ITT and 7 in PP. CBM588 was well tolerated, with no serious adverse events.

**Conclusion:**

CBM588 demonstrated potential to reduce colorectal adenoma recurrence in high-risk patients, supporting its role as a feasible, non-invasive preventive strategy.

## 1 Introduction

Colorectal cancer (CRC) is one of the most prevalent and deadly malignancies worldwide, often arising from adenomatous polyps through the adenoma-carcinoma sequence [1,2]. While routine colonoscopy and polypectomy reduce CRC incidence, polyp recurrence remains a significant challenge, necessitating novel preventive strategies.

The gut microbiota plays a pivotal role in CRC development by modulating inflammation, immune responses, and epithelial integrity [3]. Dysbiosis is associated with increased tumorigenesis, making microbiota-targeted therapies, such as probiotics, an attractive preventive option. Supporting evidence includes a trial by Ishikawa et al., showing that *Lactobacillus casei* reduced high-risk colorectal adenoma recurrence [4], and a cohort study by Zheng et al., linking higher yogurt intake to lower colorectal adenoma risk [5]. Among probiotics, *Clostridium butyricum MIYAIRI 588* (CBM588) has garnered attention due to its unique metabolic and immunomodulatory properties.

CBM588 is an anaerobic, butyrate-producing bacterium, with butyrate serving as a key energy source for colonic epithelial cells and exerting anti-inflammatory and anti-carcinogenic effects [6]. It inhibits histone deacetylase, inducing apoptosis and reducing proliferation in aberrant epithelial cells, while also enhancing intestinal barrier function by upregulating tight junction proteins and reducing gut permeability [7,8].

Beyond its metabolic effects, CBM588 modulates the immune system by promoting regulatory T cell (Treg) differentiation and reducing pro-inflammatory cytokines, creating an anti-inflammatory gut environment [9,10]. Recent studies have revealed CBM588’s potential to reshape the immune microenvironment, enhancing the efficacy of immune checkpoint inhibitors by modulating gut-specific RORγt+Tregs [11]. Additionally, CBM588 inhibits oncogenic pathways by destabilizing MYC, a transcription factor involved in CRC progression, and downregulates thymidylate synthase, enhancing sensitivity to chemotherapeutic agents like 5-fluorouracil [12]. These findings position CBM588 as a promising candidate for CRC prevention, particularly for high-risk populations.

Although preclinical studies suggest that CBM588 might modulate pathways relevant to colorectal tumorigenesis, no prospective human trial has assessed its role in preventing the recurrence of colorectal adenomatous polyps. This study aimed to fill this gap by evaluating the efficacy of CBM588 in reducing adenoma recurrence in a high-risk population. We conducted a two-year, randomized, open-label, crossover trial in patients with a history of adenomas to determine whether CBM588 could serve as an effective strategy for colorectal cancer prevention.

## 2 Material and Methods

### 2.1 Study Design and Participants

**Figure 1:**
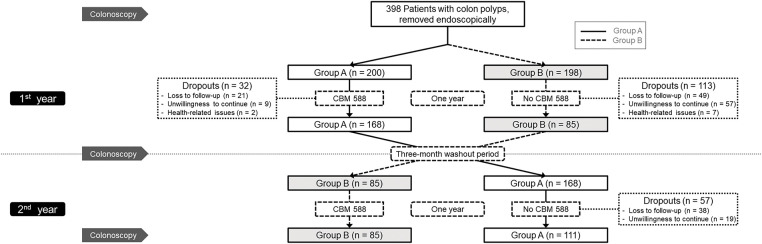
Flow diagram of the study. CBM, *Clostridium butyricum MIYAIRI*

This randomized, single-blind, crossover clinical trial enrolled 398 adult participants from Kaohsiung Medical University Hospital, Kaohsiung, Taiwan, who had a history of colorectal adenomas that had been completely removed by endoscopic resection within the past three years and subsequently presented with recurrent colorectal polyps detected during surveillance colonoscopy. Eligible participants were required to be willing to undergo scheduled follow-up colonoscopies and to adhere to the CBM588 administration protocol as specified in the study design. To ensure a homogeneous study population, individuals with a history of gastrointestinal malignancy, inflammatory bowel disease, or familial adenomatous polyposis were excluded, while participants who had taken antibiotics or probiotics within the three months prior to enrollment were also deemed ineligible. Written informed consent was obtained from all participants prior to study initiation. Participants who failed to comply with the study protocol were withdrawn from the study. The design and reporting of this trial followed the CONSORT (Consolidated Standards of Reporting Trials) guidelines for randomized controlled trials. The overall study design, including participant screening, randomization, intervention sequence, and follow-up, is illustrated in Fig. 1. This trial was approved by the Institutional Review Board of Kaohsiung Medical University Hospital (KMUHIRB-F(I)-20170010) and registered at ClinicalTrials.gov (Identifier: NCT06855355). All authors had access to the study data and approved the final manuscript.

The primary outcome of the study was to evaluate the effect of CBM588 on the recurrence of colorectal adenomatous polyps, as determined by annual colonoscopic examinations. The secondary outcome was to assess its impact on the number of recurrent colorectal polyps identified during follow-up. The sample size calculation was based on detecting a 20% absolute reduction in adenoma recurrence between treatment and control periods, assuming a two-sided alpha of 0.05 and a power of 80%. This yielded a required sample size of 100 participants, accounting for potential dropouts.

### 2.2 Intervention and Randomization

Participants were randomly assigned in a 1:1 ratio to either Group A or Group B using a computer-generated randomization list. Group A received *Clostridium butyricum MIYAIRI 588* (CBM588; MIYARISAN®BM, Miyarisan Pharmaceutical Co., Ltd., Tokyo, Japan) at a dose of 1 g per packet (containing 40 mg of CBM588 per gram), taken orally twice daily (morning and evening) for the first year, followed by a three-month washout period and no treatment in the second year. In contrast, Group B did not receive CBM588 or any placebo during the first year but began the same probiotic regimen in the second year following an identical three-month washout. The three-month washout period was implemented to allow normalization of gut microbiota and immune responses based on prior findings that probiotic effects on microbial composition and host immunity typically subside within 8 to 12 weeks [6,13]. This duration ensured a return to baseline microbial composition and intestinal epithelial turnover before crossover, minimizing residual effects from the first-year intervention [14]. A placebo was not incorporated in this study due to both ethical and logistical considerations. The crossover design was intentionally selected to ensure that all participants would receive the active intervention during the trial, aligning with ethical principles. In addition, the development and procurement of a fully inert placebo indistinguishable from CBM588 in terms of appearance, taste and formulation presented substantial practical challenges under the constraints of available resources; consequently, a single-blind design with a no-treatment control phase was employed as a feasible and ethically acceptable alternative.

### 2.3 Procedures

All colonoscopies were performed by three experienced senior gastroenterologists, each with more than 10 years of endoscopic practice, individual adenoma detection rates exceeding 25%, and average colonoscopy withdrawal times greater than 6 min. To ensure methodological rigor, all endoscopists were blinded to group allocation, maintaining a single-blind study design. Participants underwent standard bowel preparation before each procedure to optimize mucosal visualization and received reminder phone calls three days prior to each scheduled colonoscopy to enhance compliance and procedural readiness. During colonoscopy, all visible polyps regardless of size, morphology or suspected histology were completely resected. The number, histologic type (adenomatous or non-adenomatous) and size of all detected polyps were recorded at baseline, at the end of the first year, and again at the end of the second year. Throughout the study, participants were monitored for treatment adherence, adverse events, and recurrence of polyps. Demographic data, procedural outcomes and safety parameters were also collected to support comprehensive outcome analysis. Safety events were scored based on severity using a 0–10 scale, where 0 indicated no adverse event and 10 represented the most severe event, allowing for standardized evaluation and comparison of treatment-related tolerability.

### 2.4 Statistical Analysis

Data were analyzed using Stata version 15 (StataCorp, College Station, TX, USA). Both intention-to-treat (ITT) and per-protocol (PP) analyses were performed. Missing values in the ITT population were handled using multiple imputation. The imputation model included “time” and “treatment group” as predictors and assumed a missing-at-random mechanism, meaning that missingness could be explained by observed variables. Estimates of carryover effect for continuous outcomes (polyp number and size) were derived using generalized estimating equations (GEE) assuming a Gaussian distribution and exchangeable correlation structure. For categorical outcomes (polyp type and location), GEE models with binomial or multinomial logistic regression were applied as appropriate. All models were adjusted for potential confounders, including age, smoking status, alcohol consumption, and body mass index (BMI). Chi-square tests were used for categorical comparisons, and paired *t*-tests for within-group continuous data. Tests of proportion assessed changes in adenoma prevalence across time points. To evaluate clinical significance, the number needed to treat (NNT) was calculated using the formula NNT = 1/(risk control − risk treatment), based on the difference in adenoma incidence between periods with and without CBM588 use over a one-year timeframe. Results are presented with 95% confidence intervals (CIs), and statistical significance was defined as a two-sided *p*-value <0.05. A sensitivity analysis was also performed to assess the robustness of the findings by comparing baseline characteristics and outcomes between participants who completed the study and those who withdrew.

## 3 Results

From 2017 to 2024, a total of 398 patients with endoscopically removed colorectal polyps were enrolled and randomized into two groups, with 200 in Group A and 198 in Group B. During the first year, 32 participants in Group A and 113 in Group B dropped out, resulting in 168 participants completing the first year in Group A and 85 in Group B. In the second year, 57 participants from Group A did not complete follow-up, leaving 111 who completed the full two-year protocol. All 85 participants in Group B completed both years of follow-up. Ultimately, 196 participants completed all three scheduled colonoscopies and were included in the per-protocol analysis. The detailed participant distribution and dropout information are shown in Fig. 1.

### 3.1 Baseline Demographic and Clinical Characteristics

At baseline, the ITT population consisted of 200 participants in Group A and 198 in Group B. The mean age in Group A was significantly higher than in Group B (62.34 ± 0.66 vs. 59.75 ± 0.61 years, *p* < 0.01). Sex distribution was balanced between groups (male: 52.5% vs. 52.0%, *p* = 0.92). No significant differences were found in smoking (25.0% vs. 20.7%, *p* = 0.31), alcohol consumption (19.0% vs. 16.7%, *p* = 0.54), or BMI (24.18 ± 0.24 vs. 24.00 ± 0.25 kg/m^2^, *p* = 0.59).

In the PP population (Group A: n = 168; Group B: n = 85), the age difference remained significant (62.25 ± 0.70 vs. 59.11 ± 0.93 years, *p* < 0.05). Sex, smoking status, alcohol use and BMI were also similar between groups.

Regarding baseline polyp characteristics, the proportion of participants with adenomatous polyps was 71.00% in Group A and 66.67% in Group B in the ITT population (*p* = 0.35), and 71.43% vs. 61.18% in the PP population (*p* = 0.10), although differences were not statistically significant. The mean number of polyps was comparable between groups (ITT: 1.96 ± 0.10 vs. 1.92 ± 0.11, *p* = 0.76; PP: 2.01 ± 0.12 vs. 2.07 ± 0.19, *p* = 0.76). Distribution of polyp burden (≤ 1 vs. > 1) and mean polyp size (ITT: 0.48 ± 0.02 vs. 0.50 ± 0.05 cm, *p* = 0.66; PP: 0.47 ± 0.02 vs. 0.47 ± 0.04 cm, *p* = 0.94) was also balanced across groups.

Polyp location at baseline was well balanced between the two groups, with similar proportions of proximal, distal, and bilateral distribution in both ITT and PP analyses. No statistically significant differences were observed.

These comparable baseline demographic and clinical features suggest effective randomization and group comparability, as detailed in Tables 1 and 2.

**Table 1:** Demographic and clinical characteristics of study groups

Characteristics	ITT			PP		
	Group A (n = 200)	Group B (n = 198)	*p* value	Group A (n = 168)	Group B (n = 85)	*p* value
**Age (mean ± SE)**	62.34 ± 0.66	59.75 ± 0.61	<0.01	62.25 ± 0.70	59.11 ± 0.93	<0.05
**Sex**			0.92			0.25
Male	105 (52.50%)	103 (52.02%)		90 (53.57%)	39 (45.88%)	
Female	95 (47.50%)	95 (47.98%)		78 (46.43%)	46 (54.12%)	
**Smoking**	50 (25.00%)	41 (20.71%)	0.31	40 (23.81%)	18 (21.18%)	0.64
**Alcohol consumption**	38 (19.00%)	33 (16.67%)	0.54	33 (19.64%)	14 (16.47%)	0.54
**BMI**	24.18 ± 0.24	24.00 ± 0.25	0.59	24.17 ± 0.26	24.28 ± 0.38	0.81

Note: BMI, body mass index; ITT, intention-to-treat; PP, per-protocol; SE, standard error.

**Table 2:** Characteristics and colon polyp recurrence between Group A and Group B

Variables	Baseline						First year						Second year					
	ITT			PP			ITT			PP			ITT			PP		
	Group A (n = 200)	Group B (n = 198)	*p* value	Group A (n = 168)	Group B (n = 85)	*p* value	Group A (n = 200)	Group B (n = 198)	*p* value	Group A (n = 168)	Group B (n = 85)	*p* value	Group A (n = 200)	Group B (n = 198)	*p* value	Group A (n = 111)	Group B (n = 85)	*p* value
**CBM588 use**							+	−		+	–		−	+		−	+	
**Polyp type**			0.35			0.10			0.23			<0.05			0.16			0.38
Adenoma	142 (71.00%)	132 (66.67%)		120 (71.43%)	52 (61.18%)		60 (30.00%)	70 (35.35%)	0.26*	50 (29.76%)	38 (44.71%)	<0.05*	48 (24.00%)	46 (23.23%)	0.86*	28 (25.23%)	25 (29.41%)	0.51*
Non-adenoma	58 (29.00%)	66 (33.33%)		48 (28.57%)	33 (38.82%)		27 (13.50%)	33 (16.67%)		24 (14.29%)	12 (14.12%)		29 (14.50%)	17 (8.59%)		21 (18.92%)	10 (11.76%)	
No polyp							113 (56.50%)	95 (47.98%)		94 (55.95%)	35 (41.18%)		123 (61.50%)	135 (68.18%)		62 (55.86%)	50 (58.82%)	
**Polyp number (mean ± SE)**	1.96 ± 0.10	1.92 ± 0.11	0.76	2.01 ± 0.12	2.07 ± 0.19	0.76	0.78 ± 0.09	1.00 ± 0.10	0.10	0.80 ± 0.10	1.25 ± 0.16	<0.05	0.84 ± 0.10	0.95 ± 0.10	0.42	0.84 ± 0.11	0.82 ± 0.14	0.94
≤1 (median)	103 (51.50%)	109 (55.05%)	0.48	87 (51.79%)	43 (50.59%)	0.86	154 (77.00%)	137 (69.19%)	0.08	94 (55.95%)	35 (41.18%)	<0.05	134 (67.00%)	130 (65.66%)	0.78	62 (55.86%)	50 (58.82%)	0.68
>1	97 (48.50%)	89 (44.94%)		81 (48.21%)	42 (49.41%)		46 (23.00%)	61 (30.80%)		74 (44.05%)	50 (58.82%)		66 (33.00%)	68 (34.34%)		49 (44.14%)	35 (41.18%)	
**Polyp size, cm (mean ± SE)**	0.48 ± 0.02	0.50 ± 0.05	0.66	0.47 ± 0.02	0.47 ± 0.04	0.94	0.48 ± 0.03	0.43 ± 0.03	0.31	0.44 ± 0.03	0.50 ± 0.08	0.48	0.47 ± 0.03	0.41 ± 0.03	0.11	0.45 ± 0.04	0.41 ± 0.04	0.51
**Polyp location**			0.56			0.54			0.91			0.15			0.14			0.37
Proximal colon	66 (33.00%)	68 (34.34%)		57 (33.93%)	33 (38.82%)		32 (16.00%)	35 (17.68%)		28 (16.67%)	17 (20.00%)		18 (9.00%)	26 (13.13%)		12 (10.81%)	10 (11.76%)	
Distal colon	89 (44.50%)	94 (47.48%)		74 (44.05%)	38 (44.71%)		36 (18.00%)	39 (19.70%)		29 (17.26%)	22 (25.88%)		42 (21.00%)	26 (13.13%)		27 (24.32%)	13 (15.29%)	
Both sides	45 (22.50%)	36 (18.18%)		37 (22.02%)	14 (16.47%)		20 (10.00%)	20 (10.10%)		17 (10.12%)	11 (12.94%)		19 (9.50%)	22 (11.11%)		10 (9.01%)	12 (14.12%)	
No polyp							112 (56.00%)	104 (52.53%)		94 (55.95%)	35 (41.18%)		121 (60.50%)	124 (62.63%)		62 (55.86%)	50 (58.82%)	

Note: CBM, *Clostridium butyricum MIYAIRI*; ITT, intention-to-treat; PP, per-protocol; SE, standard error; *divided into two groups comprising adenoma and non-adenoma with no polyp; +, participants who received CBM588 treatment; −, participants without CBM588 treatment.

### 3.2 First Year Results

After the first year of intervention, the ITT analysis revealed a lower mean polyp number in Group A (receiving CBM588) than in Group B (0.78 ± 0.09 vs. 1.00 ± 0.10); however, the difference did not reach statistical significance (*p* = 0.10). In contrast, the PP analysis demonstrated a significantly greater reduction in mean polyp number in Group A (0.80 ± 0.10) compared to Group B (1.25 ± 0.16; *p* < 0.05). Group A had a higher proportion of participants without polyps at follow-up compared to Group B (ITT: 56.50% vs. 47.98%; PP: 55.95% vs. 41.18%). Similarly, the percentage of participants with ≤1 polyp was greater in Group A than in Group B (ITT: 77.00% vs. 69.19%, *p* = 0.08; PP: 55.95% vs. 41.18%, *p* < 0.05).

Regarding adenoma recurrence, 30.00% of participants in Group A and 35.35% in Group B developed adenomas under ITT analysis (*p* = 0.26), while PP analysis showed a statistically significant reduction in adenoma occurrence in Group A (29.76%) compared to Group B (44.71%; *p* < 0.05). Polyp size remained similar between groups and was not significantly different in either ITT (0.48 ± 0.03 cm vs. 0.43 ± 0.03 cm, *p* = 0.31) or PP (0.44 ± 0.03 cm vs. 0.50 ± 0.08 cm, *p* = 0.48) analysis.

Both groups showed a reduction in polyp presence across all colonic regions. While the distribution of proximal and distal polyps remained comparable between groups, no significant intergroup differences in location were noted, as shown in Table 2.

### 3.3 Second Year Results

Following crossover in the second year, Group B began receiving CBM588, while Group A discontinued treatment. The ITT analysis showed no significant difference in mean polyp number between Groups A and B (0.84 ± 0.10 vs. 0.95 ± 0.10, *p* = 0.42); likewise, in the PP population, mean polyp numbers were nearly identical (0.84 ± 0.11 in Group A vs. 0.82 ± 0.14 in Group B, *p* = 0.94). The percentage of participants with no polyps was similar in both groups by the end of the second year (ITT: 68.18% in Group B vs. 61.50% in Group A; PP: 58.82% vs. 55.86%, *p* = 0.68). Similarly, the proportion of participants with ≤1 polyp was consistent across groups (ITT: 67.00% vs. 65.66%, *p* = 0.78; PP: 55.86% vs. 58.82%, *p* = 0.68).

In terms of adenoma prevalence, Group A and Group B exhibited nearly equal rates in the ITT analysis (24.00% vs. 23.23%, *p* = 0.16), and no significant difference was observed in the PP analysis either (25.23% vs. 29.41%, *p* = 0.38). Polyp size also remained similar between groups (ITT: 0.47 ± 0.03 cm vs. 0.41 ± 0.03 cm, *p* = 0.11; PP: 0.45 ± 0.04 cm vs. 0.41 ± 0.04 cm, *p* = 0.51).

By the end of the second year, the majority of participants were polyp-free, and the distribution of remaining polyps showed no significant difference in location between groups. The reductions appeared consistent across both proximal and distal sites following CBM588 treatment, as shown in Table 2.

### 3.4 Trends of Polyp Number over Time

**Figure 2:**
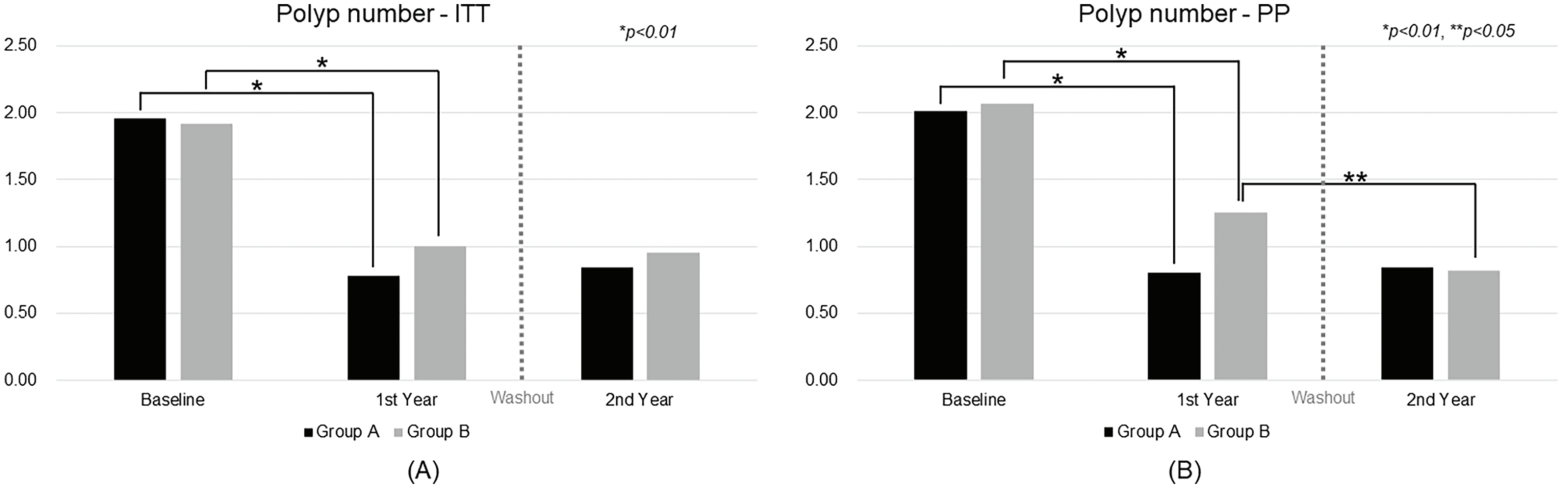
Trend analysis of polyp number over time. ITT, intention-to-treat; PP, per-protocol

In the ITT analysis (Fig. 2A), both groups showed significant reductions in polyp number following CBM588 treatment. Group A’s mean polyp count decreased from 1.96 at baseline to 0.78 after the first year (*p* < 0.01), and remained low at 0.84 in the second year. However, the difference between year 1 and year 2 was not statistically significant (*p* = 0.09). Group B also exhibited a significant decline from 1.92 at baseline to 1.00 in the first year (*p* < 0.01) and to 0.95 in the second year, with no significant change between the two years (*p* = 0.22).

In the PP analysis (Fig. 2B), similar patterns were observed. Group A’s polyp number dropped from 2.01 at baseline to 0.80 in the first year (*p* < 0.01) and remained stable at 0.84 in the second year. Group B’s count declined from 2.07 to 1.25 in the first year (*p* < 0.01) and further to 0.82 in the second year, with a significant reduction between the two years (*p* = 0.02).

### 3.5 Adenoma Proportion Changes over Time

**Figure 3:**
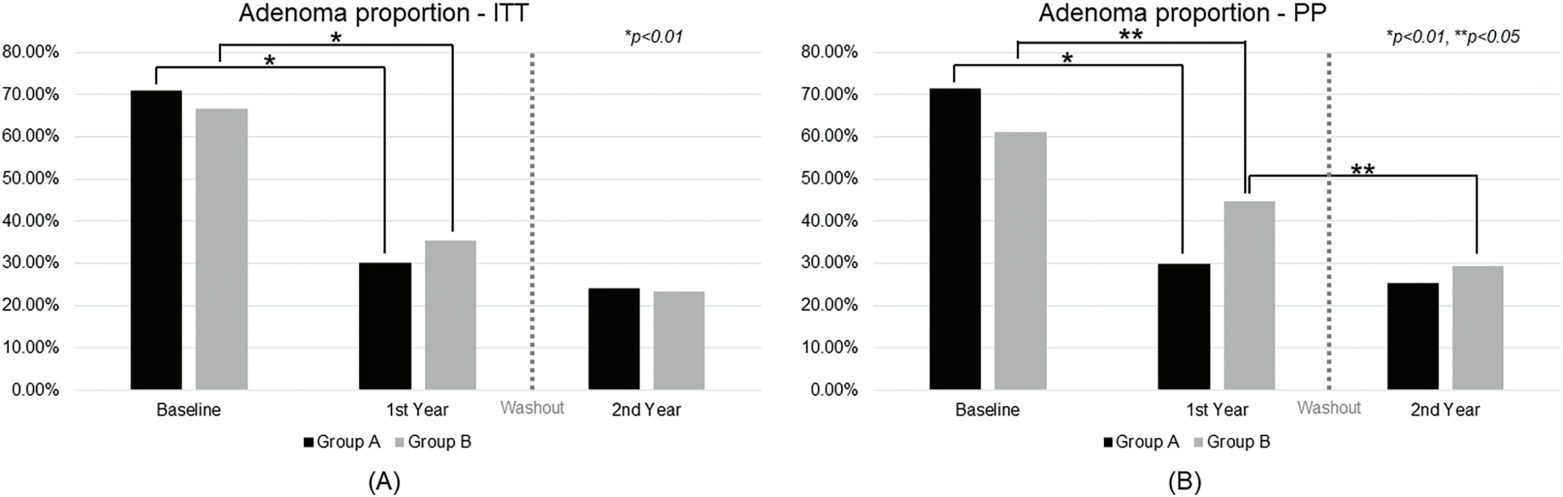
Trend analysis of adenoma proportion over time. ITT, intention-to-treat; PP, per-protocol

As shown in Fig. 3A, ITT analysis revealed that both groups experienced significant reductions in adenoma prevalence during CBM588 treatment. In Group A, the adenoma rate declined from 71.00% at baseline to 30.00% after the first year (*p* < 0.01) and remained low at 24.0% in the second year. The difference between year 1 and year 2 was not statistically significant (*p* = 0.26). Group B showed similar improvement, with adenoma prevalence decreasing from 66.67% at baseline to 35.35% in the first year (*p* < 0.01) and further to 23.23% in the second year, also without a significant change between the two years (*p* = 0.10). Based on these results, the NNT to prevent recurrence over one year in the ITT population was estimated to be 19, indicating a moderate clinical benefit.

In the PP analysis (Fig. 3B), the effect was more pronounced. Group A dropped from 71.43% to 29.76% in the first year (*p* < 0.01) and to 25.23% in the second year, with no significant change between the two years (*p* = 0.41). Group B declined from 61.18% to 44.71% in the first year (*p* < 0.05) and further to 29.41% in the second year, with a statistically significant reduction between the two years (*p* < 0.05). The corresponding NNT in the PP population was 7, suggesting a more substantial preventive effect among participants who adhered to the treatment protocol.

### 3.6 Carryover Effect Analysis

To evaluate potential carryover effects, treatment outcomes during the CBM588 administration periods were compared across the two study sequences. No significant differences were found in polyp number or size. Similarly, polyp location and histological type showed comparable distributions across treatment periods. These findings, as shown in Table 3, were consistent in both the ITT and PP populations, suggesting minimal carryover effects and supporting the adequacy of the washout period.

**Table 3:** Carryover effect analysis between treatment sequences

Outcomes	ITT			PP		
	β	95% CI	*p* value	β	95% CI	*p* value
**Polyp number**	−0.32	−0.73–0.08	0.12	−0.48	−1.04, −0.07	0.09
**Polyp size**	0.11	−0.01–0.23	0.07	0.05	−0.11, 0.21	0.54
	OR	95% CI	*p* value	OR	95% CI	*p* value
**Polyp type**						
Adenoma	0.83	0.42–1.64	0.58	0.44	0.17–1.14	0.09
Non-adenoma	1.29	0.52–3.19	0.59	1.26	0.38–4.18	0.70
**Polyp location**						
Proximal colon	0.60	0.25–1.46	0.26	0.59	0.16–2.16	0.43
Distal colon	1.42	0.67–2.98	0.36	0.82	0.29–2.32	0.71
Both sides	0.82	0.31–2.19	0.69	0.39	0.10–1.50	0.17

Note: Estimates of carryover effect for continuous outcomes (polyp number and size) were derived using GEE assuming a Gaussian distribution and exchangeable correlation. Categorical outcomes (polyp type and location) were analyzed using GEE with binomial or multinomial logistic regression. All models were adjusted for age, smoking, alcohol consumption and BMI. Results are presented for both ITT and PP; β, beta coefficient; BMI, body mass index; CI, confidence interval; GEE, generalized estimating equations; ITT, intention-to-treat; OR, odds ratio; PP, per-protocol.

### 3.7 Adverse Events and Safety Profile

As summarized in Table 4, CBM588 was generally well tolerated, with no serious adverse events reported during the study. All adverse events were self-limited and resolved without the need for medical intervention. The majority of events scored ≤3, indicating mild severity. No significant differences in adverse event rates were observed between groups. These findings suggest that long-term administration of CBM588 is safe and well tolerated.

**Table 4:** Summary of adverse events and symptom severity during CBM588 administration

Adverse events	ITT			PP		
	Group A (n = 200)	Group B (n = 198)	*p* value	Group A (n = 111)	Group B (n = 85)	*p* value
**Abdominal pain**	1.06 ± 0.12	1.03 ± 0.12	0.87	1.09 ± 0.13	1.31 ± 0.20	0.35
**Diarrhea**	0.59 ± 0.08	0.51 ± 0.09	0.55	0.57 ± 0.09	0.71 ± 0.18	0.44
**Constipation**	0.71 ± 0.12	0.56 ± 0.10	0.32	0.66 ± 0.12	0.41 ± 0.10	0.17
**Tenesmus**	1.33 ± 0.14	1.00 ± 0.12	0.08	1.27 ± 0.15	1.26 ± 0.22	0.96
**Bloating**	0.92 ± 0.13	0.76 ± 0.10	0.30	0.85 ± 0.13	0.77 ± 0.16	0.70
**Belching**	2.01 ± 0.16	1.74 ± 0.15	0.22	1.89 ± 0.17	1.69 ± 0.23	0.48
**Reflux**	1.89 ± 0.16	1.72 ± 0.16	0.47	1.87 ± 0.18	1.95 ± 0.24	0.78

Note: Symptom severity was rated on a 0–10 scale, with 0 indicating none and 10 the most severe; ITT, intention-to-treat; PP, per-protocol.

### 3.8 Sensitivity Analysis and Robustness Assessment

To evaluate the robustness of our findings, we conducted a sensitivity analysis comparing baseline characteristics between participants who completed the study (completers) and those who withdrew (dropouts). As summarized in Table 5, there were no significant differences between the two groups across key demographic and clinical variables, including age, sex, BMI, smoking, alcohol use, adverse events, baseline adenoma number, and size. These findings suggest minimal selection bias due to dropout and support the generalizability of the study results.

**Table 5:** Comparison of baseline characteristics and adverse events between completers and dropouts

Variables	Completers (n = 196)	Dropouts (n = 202)	*p* value
**Age (mean ± SE)**	61.20 ± 0.57	60.79 ± 0.76	0.66
**Sex**			0.50
Male	96 (48.98%)	110 (54.46%)	
Female	100 (51.02%)	92 (45.54%)	
**Smoking**	45 (22.96%)	46 (22.77%)	0.97
**Alcohol consumption**	36 (18.37%)	33 (16.33%)	0.61
**BMI**	24.21 ± 0.22	23.89 ± 0.28	0.37
**Polyp type**			0.63
Adenoma	133 (67.86%)	142 (70.30%)	
Non-adenoma	63 (32.14%)	60 (29.70%)	
**Polyp number**	2.01 ± 0.10	1.83 ± 0.10	0.23
**Polyp size, cm**	0.47 ± 0.02	0.53 ± 0.06	0.27
**Adverse events**			
Abdominal pain	1.16 ± 0.11	0.85 ± 0.12	0.07
Diarrhea	0.61 ± 0.08	0.44 ± 0.09	0.19
Constipation	0.57 ± 0.08	0.75 ± 0.15	0.27
Tenesmus	1.27 ± 0.12	0.98 ± 0.14	0.15
Bloating	0.82 ± 0.10	0.87 ± 0.13	0.78
Belching	1.82 ± 0.14	1.97 ± 0.19	0.52
Reflux	1.90 ± 0.14	1.65 ± 0.20	0.31

Note: BMI, body mass index; SE, standard error.

## 4 Discussion

### 4.1 Efficacy of CBM588 in Reducing Adenoma Recurrence

This randomized crossover trial provides evidence that the probiotic CBM588 showed a potential benefit in reducing the recurrence of colorectal adenomatous polyps. Both treatment sequences showed notable decreases in polyp number and adenoma count during CBM588 administration, suggesting a clear probiotic effect. The pronounced decline in Group B after crossover supports that the benefit was attributable to the probiotic rather than natural history. These findings align with a growing body of evidence that modulating the gut microbiome with probiotics can favorably influence colorectal neoplasia risk by creating a less pro-carcinogenic intestinal environment [15]. Notably, polyps detected during the second and third annual colonoscopies were small (≤0.5 cm) and were likely that the majority of these small polyps represented new (metachronous) lesions rather than lesions missed on prior examinations. This was supported by the high quality of colonoscopy throughout the study, with experienced endoscopists, adequate bowel preparation, and standardized techniques. While the possibility of missing small, flat, or sessile serrated polyps could not be entirely excluded, the majority of later polyps were likely *de novo* lesions, highlighting the need for continued surveillance, as new adenomas might still arise despite effective probiotic therapy.

The clinical relevance of our findings is supported by the NNT, estimated at 19 in the ITT and 7 in the PP population, to prevent one recurrence over one year. All recurrent lesions detected in our study were diminutive and identified at an early stage, supporting CBM588 as a promising non-invasive strategy to reduce adenoma recurrence and potentially lower long-term colorectal cancer risk. However, the higher dropout rate in Group B warrants caution. Although dropout analyses revealed no significant baseline differences, the possibility remains that individuals at lower risk were more likely to stay in the study, potentially amplifying the observed treatment effect.

### 4.2 Clinical Relevance in a High-Risk Population

An important clinical feature of this study was its focus on a high-risk population. All participants had a mean age over 45 years, with documented adenomas removed within the past three years and recurrent adenomas confirmed at enrollment. This profile is consistent with patients at elevated risk for metachronous neoplasia [16]. Although there was a statistically significant difference in age between groups, the difference was relatively small and unlikely to have materially influenced the study outcomes, especially given the consistent treatment effects observed across both groups. Consequently, the relatively high adenoma detection rates in the second and third surveillance colonoscopies were expected. Moreover, the decision to conduct colonoscopy at annual intervals was clinically appropriate and aligns with U.S. Multi-Society Task Force guidelines, which recommend shorter surveillance intervals for high-risk patients [17].

### 4.3 Biological Mechanisms Underpinning CBM588’s Effect

CBM588’s protective effect is biologically plausible due to its production of butyrate, a key short-chain fatty acid that maintains colonic homeostasis [6]. Butyrate has well-established anti-neoplastic properties as it promotes the differentiation and apoptosis of colonocytes while inhibiting aberrant proliferation, partly through epigenetic mechanisms such as histone deacetylase inhibition [18]. This modulation may restore tumor-suppressor pathways and limit early neoplastic changes. Butyrate has also been shown to suppress the growth of preneoplastic colon adenoma cells more effectively than carcinoma cells [18], suggesting a role in early-stage prevention. In our study, however, intestinal butyrate levels were not directly measured, and this mechanistic link remains speculative.

Butyrate also reinforces the gut barrier by enhancing tight junction proteins (e.g., claudins, occludin), improving epithelial integrity and reducing the translocation of pro-inflammatory microbes or toxins [19,20]. A stronger barrier mitigates endotoxin-induced chronic inflammation, which is relevant to colorectal tumorigenesis. Additionally, CBM588 favorably remodels the gut microbiota, increasing beneficial genera such as *Lactobacillus* and *Bifidobacterium*, while reducing pathogenic bacteria [21,22]. These changes are associated with decreased levels of carcinogenic metabolites such as secondary bile acids and N-nitrosamines [22–24].

Beyond metabolic and microbial effects, CBM588 also exerts immune modulation. Microbial-derived butyrate can induce colonic Tregs [25], which dampen pro-inflammatory responses by suppressing cytokines such as IL-6 and TNF-α. These cytokines are known to drive epithelial proliferation and angiogenesis in CRC [10,12,26]. Butyrate exposure has been shown to reduce their production by immune cells, creating a tumor-suppressive microenvironment [26].

Collectively, these multifaceted metabolic, epithelial, microbial, and immune actions offer a biologically plausible rationale for the observed reduction in colorectal adenoma recurrence. Although the proposed mechanisms are supported by prior evidence, they remain hypothetical in the context of this study.

### 4.4 Limitations

#### 4.4.1 High Dropout Rate and Participant Attrition

The relatively high dropout rate, particularly in Group B during the first year, may have introduced bias. Although the crossover design and ITT analysis help mitigate this, differential attrition could affect group comparability. A likely reason for the higher attrition in Group B is that participants did not receive CBM588 during the first year, which might have led to reduced motivation and engagement. Without an active intervention or placebo control, participants might well have perceived a lack of benefit and thus opted to discontinue. Additionally, the demanding protocol which required daily intake of CBM588 for a full year along with three scheduled colonoscopies over the study period likely contributed to follow-up fatigue, discomfort with repeated endoscopic procedures as well as logistical challenges, especially among older participants or those with other comorbidities. Such intensive regimens are well-documented to reduce long-term adherence [27,28]. Common reasons for dropout included follow-up fatigue, discomfort with repeated colonoscopy, and unrelated health issues.

#### 4.4.2 Insufficient Duration

The two-year intervention with three colonoscopies might be insufficient to fully assess the long-term durability of CBM588’s protective effect on colorectal neoplasia. While adenoma recurrence is a widely accepted surrogate endpoint, a longer follow-up period is necessary to evaluate sustained benefit, particularly regarding colorectal cancer risk reduction. Moreover, the study design did not include intermediate assessments, which could have helped identify the timing and persistence of CBM588’s effect. Although crossover results suggest a residual benefit one year after cessation of treatment, further follow-up at multiple time points is warranted to better understand the duration and trajectory of efficacy.

#### 4.4.3 Potential Uncontrolled Confounders and Lack of Subgroup Analysis

Although baseline characteristics including sex, BMI and adenoma burden appeared balanced between the treatment sequences, residual confounding remains a concern, as some potentially important variables such as diet, physical activity and over-the-counter NSAID use were not fully captured or adjusted for. These factors are known to influence colorectal polyp recurrence [29], and their unmeasured effects may limit the internal validity of our findings.

In addition, while exploratory subgroup stratifications were conducted (e.g., by age, sex and baseline adenoma number), no consistent treatment effect differences were observed, although the study was not adequately powered to detect interactions and no pre-specified subgroup analyses were performed. These limitations restrict our ability to identify whether certain patient populations would derive greater benefit from CBM588. Future studies with larger sample sizes should include predefined stratified analyses and collect more comprehensive baseline data to allow for better adjustment of confounding and to enhance the generalizability of results.

#### 4.4.4 Absence of Microbiome and Immunologic Analyses

The study did not include direct assessment of gut microbiota composition or immune biomarkers. Although prior studies suggest that *Clostridium butyricum* could modulate the gut microbiota and promote anti-inflammatory immune responses such as inducing IL-10–producing macrophages and enhancing tumor immunity [30,31], we were unable to measure these mechanisms in this study. Future trials should incorporate microbiome and immunologic profiling to better elucidate the link between CBM588 and its clinical effects.

#### 4.4.5 Lack of Placebo Control and Risk of Behavioral Bias

This study used an open-label design without a placebo, which may have introduced behavioral bias, as participants knew their treatment group. Although endoscopists were blinded and standardized procedures were followed to reduce observer bias, the lack of a double-blind, placebo-controlled design may limit internal validity. For example, participants who were aware they were not receiving CBM588 during the control phase may have been less motivated to adhere to study protocols, or may have reported gastrointestinal symptoms differently, potentially influencing outcome assessments. Future studies should adopt such a design to minimize bias and improve reliability.

## 5 Conclusion

This randomized crossover trial demonstrates that CBM588 has the potential to significantly reduce colorectal adenoma recurrence, particularly in individuals with high treatment adherence. While the reduction in mean polyp number did not reach statistical significance in the ITT analysis, the consistent and substantial decreases observed in the PP analysis underscore the clinical relevance of CBM588’s effects. These results highlight CBM588 as a promising, non-invasive strategy for adenoma prevention, especially in high-risk populations. Given its favorable safety profile and biological plausibility, further large-scale, placebo-controlled trials are warranted to confirm its preventive efficacy and to elucidate the underlying mechanisms.

## Data Availability

The data that support the findings of this study are available from the Corresponding Author, Deng-Chyang Wu, upon reasonable request.

## Abbreviations

BMI: Body mass index
CBM588: *Clostridium butyricum MIYAIRI 588*
CRC: Colorectal cancer
GEE: Generalized estimating equations
ITT: Intention-to-treat
NNT: Number needed to treat
OR: Odds ratio
PP: Per-protocol
SE: Standard error
Treg: Regulatory T cell
